# Femoral Shaft Cortical Pathology associated with longterm Alendronate Therapy: A New Classification

**DOI:** 10.5704/MOJ.1307.008

**Published:** 2013-07

**Authors:** MK Kwan, CK Chan, WM Ng, AM Merican, WM Chung, SP Chan

**Affiliations:** Department of Orthopaedic Surgery, University of Malaya, Kuala Lumpur, Malaysia; Department of Orthopaedic Surgery, University of Malaya, Kuala Lumpur, Malaysia; Department of Orthopaedic Surgery, University of Malaya, Kuala Lumpur, Malaysia; Department of Orthopaedic Surgery, University of Malaya, Kuala Lumpur, Malaysia; Department of Orthopaedic Surgery, University of Malaya, Kuala Lumpur, Malaysia; Department of Medicine, University of Malaya, Kuala Lumpur, Malaysia

## Abstract

There are reported cases of cortical reaction over the tension side of the normal femoral shafts in patients on long term treatment with alendronate, leading to subsequent femoral shaft fractures. We performed a retrospective review of patients with low-energy femoral shaft fracture on alendronate, admitted to our institution during the period 2004 to May 2009. The presence of radiological changes of cortical hypertrophy with or without Looser’s zone over the tension side of the femoral bone (normal limb) was determined and correlated with clinical symptoms. Thirteen patients were identified. Average duration of alendronate use was 6.5 ± 3.3 years (ranges, two to 10 years). These radiological changes were noted in four patients. Average duration of alendronate usage in these four patients was 6.5 ± 2.4 years (ranges, 5 to10 years). Prodromal thigh pain was present in a patient, who had cortical hypertrophy with the presence of a Looser’s zone traversing the cortex on the femoral shaft. One patient had Looser’s zone limited at the lateral hypertrophied cortex without prodromal pain. The interobserver kappa coefficient was 0.96. A femoral radiograph should be performed in all patients who are on long-term alendronate therapy who present with thigh pain. We propose a new grading system based on our observation of the radiological features in these four cases. This new grading of the radiological spectrum of femoral shaft cortical pathology has the potential to stratify the risk of low energy femoral fracture for patients treated with long-term alendronate therapy.

**Key Words:**

Femur, Cortical Hypertrophy, Looser Zone, Alendronate

## Introduction

Osteoporosis is an increasingly common health problem characterised by imbalance in the rate of bone resorption and formation, generally in conjunction with increased rate of bone turnover^1^. The progressive decrease in bone mass leads to increased susceptibility to fracture. Alendronate has been the most widely used anti-resorptive agent and is recommended for the prevention and treatment of postmenopausal osteoporosis. Alendronate given in the optimal dose can increase bone mineral density (BMD) of the spine and hip by 5-9% in two to three years and reduces the risk of vertebral and hip fractures by up to 50% in women with osteoporosis^2^. However, some recent studies warn that prolonged treatment with alendronate may lead to adynamic, fragile bone^3^.

As described by Goh et al.^3^, we too have observed the presence of cortical reaction over the tension side in the contra-lateral femur (normal limb) in patients treated with long-term alendronate, who presented with a femoral shaft fracture. These abnormal radiological findings raised our interest to further evaluate the significance of these radiological cortical pathology in relation to patient clinical presentation, and subsequently develop a guideline to management of this disease

## Materials and Methods

After obtaining institutional ethical committee approval, thirteen patients who presented with a low energy subtrochanteric or proximal femoral shaft fracture from 2004 to 2009 were identified. All of them agreed to participate in this study. A low energy fracture was defined as that caused by a fall from a height equal or less than the patient’s standing height. The medical records were reviewed and relevant information such as use of alendronate, prodromal pain (such as discomfort and weird unexplained aching), comorbidities and bone mineral density scan results were retrieved. We excluded patients with other causes of osteoporosis (such as treatment with glucocorticoids) or other disorders of metabolic bone disease (such as Paget’s disease or hyperparathyroidism). The patients were contacted by telephone to verify the accuracy of the documented information. Radiograph of the contra-lateral limb (non-fracture limb) were taken if there were no previous radiograph of the contra-lateral limb.

Three senior orthopaedic surgeons and an orthopaedic resident independently reviewed the radiographs of the contralateral femur of these patients on separate occasions. The radiographs were displayed in a computer-based slide presentation to each reviewer separately. The order of the radiographs was random and changed in each session. The reviewers were asked to identify the cortical hypertrophy (cortical thickening) with or without the Looser’s zone (crack line at the cortex) over the cortex of the femoral bone. Fleiss’s and Cohen’s kappa coefficients for interobserver agreement were calculated by comparing the proportion of agreement in relation to the agreement as a result of chance. Kappa values of less than 0.40 indicate poor agreement, whereas values greater than 0.81 indicate near-perfect agreement^4^.

## Results

Thirteen patients presenting with low energy femoral fractures were identified and analyzed. The average age was 70.2 ± 6.8 years (ranges, 58 to79). Twelve of the fractures were subtrochanteric, and one was located at the junction between the proximal third and middle third of the femoral shaft. All the fractures had occurred after a trivial injury, mostly slip and fall. The mean duration of alendronate use was 6.5 ± 3.3 years (ranges, 2 to10 years). The information regarding the administration of the alendronate, the BMD (whenever available) and the year of injury are shown in Table I.

The BMD results for four patients who were diagnosed to have osteoporosis by their physicians were not available. The radiographs of the contralateral femur of all these patients were reviewed by four observers independently. Four patients were identified to have radiological changes such as cortical hypertrophy over the tension side of the contralateral femoral shaft. The interobserver kappa coefficient was 0.96. One patient had prodromal pain over the thigh and the radiograph revealed cortical hypertrophy with presence of a Looser’s zone traversing the cortex. She was treated non-surgically and it healed spontaneously (Figure 1). Another patient had a small area of Looser’s zone over the cortex without presentation of prodromal pain. The remaining two patients with the cortical hypertrophy over the tension side of the femur were asymptomatic. (Figure 2, 3, 4) The average duration of alendronate usage was 6.5 ± 2.4 years (5-10 years). The other nine patients without the radiological changes were asymptomatic of prodromal pain. The alendronate was stopped for all the patients after the fractures.

Besides, we also noticed that the four patients with cortical changes over the femoral bone had bone mineral density (BMD), classified in the group of normal to osteopenia (three osteopenia and one normal) according to the World Health Organisation (WHO) working group classification of osteoporosis; whereas those who did not have the cortical changes had the BMD in group of osteopenia to osteoporosis. Nevertheless, the difference between these two groups was not significant.

Case 1. Madam LL, 64 years old, on alendronate treatment for five years (T score was -2.2) presented with prodromal pain over the left thigh for one week. Radiograph of the femur revealed hypertrophy of the tension site over the left femoral subtrochanteric region. (Figure 1A, white arrow). One week later, she sustained a fracture of her left subtrochanteric region after a trivial fall. The fracture pattern was typical: transverse fracture with cortical beaking and cortical hypertrophy (Figure 1B, white arrow). The fracture was treated with an intramedullary sliding device. Two weeks later, she developed prodromal pain over the right thigh. Radiograph of the right femur revealed the presence of cortical hypertrophy over the tension side of the right femur at the similar region (Figure 1C, white arrow). Lateral view of the right femur shows that there was presence of Looser’s zone over the cortical hypertrophy site traversing the cortex (Figure 1D, white arrow). Option for internal fixation was offered but patient requested conservative treatment. She was put on bed rest for one month and the right thigh pain resolved. Alendronate treatment was stopped. At nine months follow-up, radiographs revealed that the left subtrochanteric fracture and right pre-fracture healed uneventfully.(Figure 1)

Case 2. Madam WSM, 69-year-old, on alendronate treatment for five years (T score was -2.2) presented with left femoral shaft fracture after a trivial fall. The fracture was typical: transverse fracture with cortical beaking (Figure 2A white arrows). The fracture was treated with an interlocking nail. Contra-lateral radiograph of the right lower limb revealed the presence of cortical hypertrophy was noted at the tension side of the femur at the same region. (Figure 2B, white arrow). A small Looser’s zone was noted in the lateral cortex in high magnification. There was not clinical complaint of prodromal pain. Alendronate was stopped after the fracture. (Figure 2)

Figure 3: Case 3. Madam TAN, 70-year-old, on alendronate for 10 years (T score was -0.6), sustained a subtrochanteric fracture of the right femur after a trivial fall. Contra-lateral limb radiograph revealed the presence of cortical hypertrophy over the tension side of femur. (Figure 3B, white arrow) Otherwise, the patient did not have prodromal pain. The alendronate treatment was stopped after the fracture. (Figure 3)

Case 4. Madam LYS, 79 years old, on alendronate treatment for six years (T score was -2.4), sustained a left subtrochanteric femoral fracture which had a transverse pattern with cortical beaking (Figure 4A, white arrow) She was treated with internal fixation. Contra-lateral femoral radiograph was performed and similar to the other patients described, the presence of cortical hypertrophy over the tension side of the right femur was noted. (Figure 4B, white arrow) Otherwise, she did not have any thigh pain. The alendronate treatment was stopped after the fracture. (Figure 4)

## Discussion

Femoral diaphysial fractures occur most frequently in young patients as a result of high-energy trauma. In a recent study the bending force needed to produce a femoral shaft fracture in a normal adult has been estimated at 250 Nm, and may exceed 8000 Nm in purely axial compression^7^. However, we note the presence of low energy femoral shaft fracture in patient with osteoporosis, particularly after prolonged exposure to potent anti-resorptive therapy such as alendronate use. These fractures were thought to be rarely caused by osteoporosis per se, accounting for approximately 6% of osteoporosis fractures. Our patients sustained low energy transverse fractures of the femur, as reported by Neviaser AS et al; a simple transverse fracture, cortical beaking and cortical hypertrophy at the tension side of the fractured femur after trivial trauma^5^.

Alendronate is an anti-resorptive agent, which is a synthetic pyrophosphate analogue that binds to hydroxyapatite crystals in the bone. It inhibits osteoclast function and promotes osteoclast apoptosis^8^. Due to these potent cellular pharmacological effects; alendronate is widely used to treat osteoporosis. It is well recognised that osteoporotic fractures depend on factors such as bone density, the bone turnover rate, microarchitecture, geometry and mineralisation^9^. Although alendronate use leads to an increase in bone mineral density of patient with osteoporosis, it carries the potential risk of over-suppressing bone turnover, which ultimately may impair some of the biomechanical properties of the bone such as microdamage accumulation and may cause the bone to become more brittle. This could indirectly result in an increased risk of fracture^10^.

The unique fracture patterns as well as the cortical pathology in patients treated with alendronate, suggest that osteoporosis alone is insufficient to cause this specific failure of the femur^5^. This is supported by our findings as well as Goh et al^3^. findings that the cortical pathology and fracture occur in patients with the BMD mostly in the group of osteopenia. As such, we believe that the presence of cortical hypertrophy with or without Looser’s zone detected over the contralateral femur may imply that the quality of the bone produced is abnormal. As a result of the chronic suppression of bone turnover from impaired bone resorption, the bone becomes more brittle. This is supported by animal studies, which have displayed significant increases in microdamage following bisphophonate treatment^11^,^12^. Because of this, a new form of abnormal stress fracture over the tension side of the femoral shaft has been increasingly identified as the radiographic features of these cortical pathology share similarities with the radiographic features of fatigue or stress fractures.

Based on the observation in our review of these four cases, we note a specific radiographic spectrum of femoral cortical hypertrophy as a result of long-term alendronate treatment. Initially the stresses over the over-suppressed femur will result in cortical hypertrophy over the lateral tension side of the femoral cortex (Case 3 and 4). With time the cortical hypertrophy becomes more extensive and slowly progresses to form Looser’s zones indicating a form of chronic fatigue or stress fracture. (Case 1 and 2) As the condition progresses it will become symptomatic i.e. prodromal thigh pain and fracture if the stresses persist without intervention (Case 1). Our small series of patients add to those that have been recently reported in the literature and should raise awareness of the possibility of occurrence of this pathology of the femoral cortex. Close attention should be paid to these highrisk individuals and early intervention initiated to prevent further aggravation of this cortical pathology to fracture. Symptom of prodromal thigh pain in patients with long-term alendronate therapy should alert us to perform a radiograph of the femur so that early intervention can be instituted.

We would like to propose a simple and intuitive grading of this radiological spectrum of cortical pathology, noted over the tension side of the femur. (Table II) We have incorporated the clinical symptoms i.e. prodromal pain to emphasize the importance of this sign as it is indicative of impending fracture. The limitation of this paper is the number of cases is small.

**Table I:**
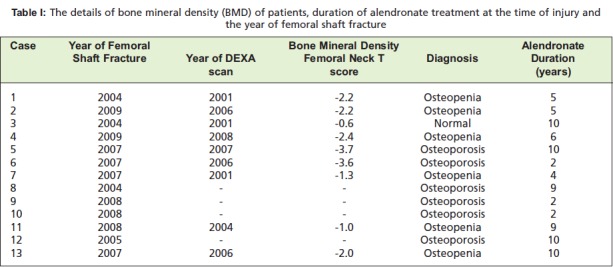
: The details of bone mineral density (BMD) of patients, duration of alendronate treatment at the time of injury and the year of femoral shaft fracture

**Table II:**
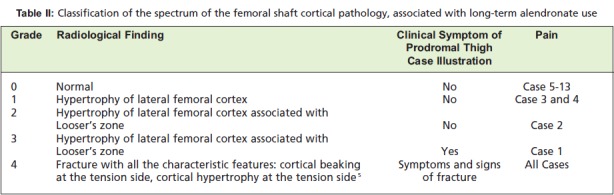
: Classification of the spectrum of the femoral shaft cortical pathology, associated with long-term alendronate use

**Fig. 1A:**
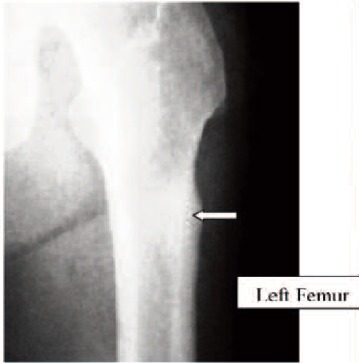
: (Pre-fracture)

**Fig. 1B:**
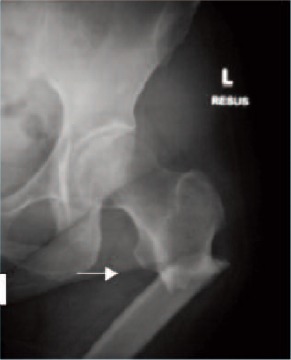
: (one week later)

**Fig. 1C:**
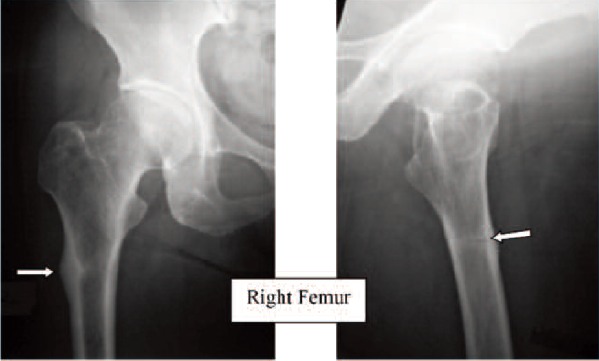
: Right Femur

**Fig. 1D:**
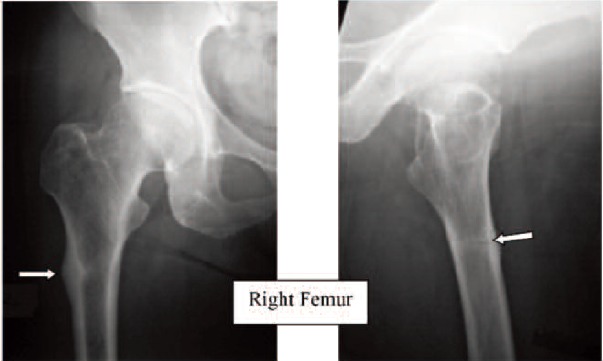
: Right Femur

**Fig. 2A:**
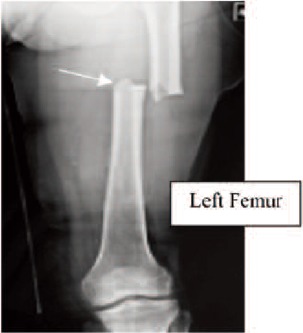
: Left Femur

**Fig. 2B:**
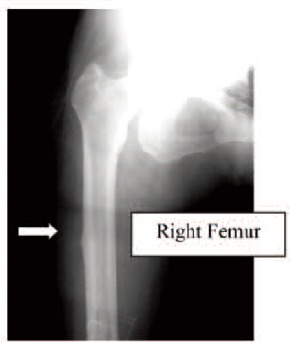
: Right Femur

**Fig. 3A:**
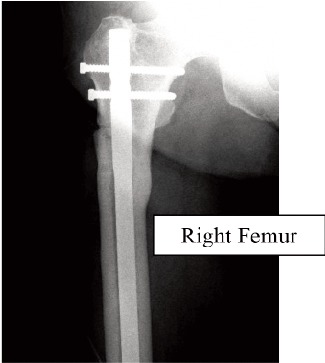
: Right Femur

**Fig. 3B:**
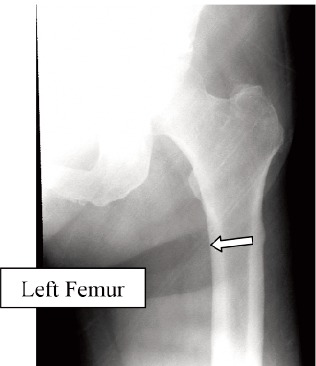
: Left Femur

**Fig. 4A:**
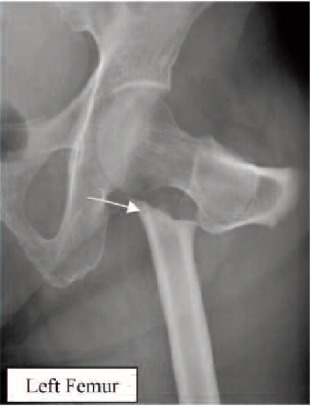
: Left Femur

**Fig. 4B:**
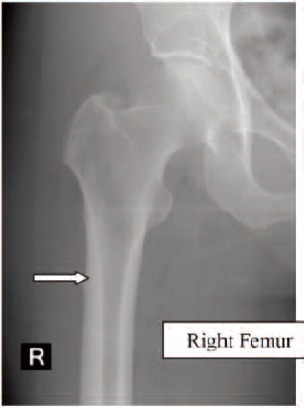
: Right Femur

## Conclusion

This new classification will help us to stratify the severity of these pathologies and may serve as a reference for the management of this condition in the future. We also hope that this proposed grading system would improve the communication of the spectrum of cortical pathologies occurring over the tension side of the femur in patients treated with long-term alendronate therapy.
